# Phylogenetic analysis of eIF4E-family members

**DOI:** 10.1186/1471-2148-5-48

**Published:** 2005-09-28

**Authors:** Bhavesh Joshi, Kibwe Lee, Dennis L Maeder, Rosemary Jagus

**Affiliations:** 1 Center of Marine Biotechnology, Suite 236 Columbus Center, 701 E. Pratt Street, Baltimore, MD 21202, USA

## Abstract

**Background:**

Translation initiation in eukaryotes involves the recruitment of mRNA to the ribosome which is controlled by the translation factor eIF4E. eIF4E binds to the 5'-m^7^Gppp cap-structure of mRNA. Three dimensional structures of eIF4Es bound to cap-analogues resemble 'cupped-hands' in which the cap-structure is sandwiched between two conserved Trp residues (Trp-56 and Trp-102 of *H. sapiens *eIF4E). A third conserved Trp residue (Trp-166 of *H. sapiens *eIF4E) recognizes the ^7^-methyl moiety of the cap-structure. Assessment of GenBank NR and dbEST databases reveals that many organisms encode a number of proteins with homology to eIF4E. Little is understood about the relationships of these structurally related proteins to each other.

**Results:**

By combining sequence data deposited in the Genbank databases, we have identified sequences encoding 411 eIF4E-family members from 230 species. These sequences have been deposited into an internet-accessible database designed for sequence comparisons of eIF4E-family members. Most members can be grouped into one of three classes. Class I members carry Trp residues equivalent to Trp-43 and Trp-56 of *H. sapiens *eIF4E and appear to be present in all eukaryotes. Class II members, possess Trp→Tyr/Phe/Leu and Trp→Tyr/Phe substitutions relative to Trp-43 and Trp-56 of *H. sapiens *eIF4E, and can be identified in Metazoa, Viridiplantae, and Fungi. Class III members possess a Trp residue equivalent to Trp-43 of *H. sapiens *eIF4E but carry a Trp→Cys/Tyr substitution relative to Trp-56 of *H. sapiens *eIF4E, and can be identified in Coelomata and Cnidaria. Some eIF4E-family members from Protista show extension or compaction relative to prototypical eIF4E-family members.

**Conclusion:**

The expansion of sequenced cDNAs and genomic DNAs from all eukaryotic kingdoms has revealed a variety of proteins related in structure to eIF4E. Evolutionarily it seems that a single early eIF4E gene has undergone multiple gene duplications generating multiple structural classes, such that it is no longer possible to predict function from the primary amino acid sequence of an eIF4E-family member. The variety of eIF4E-family members provides a source of alternatives on the eIF4E structural theme that will benefit structure/function analyses and therapeutic drug design.

## Background

The recruitment of mRNAs to the ribosomal apparatus is a key step in the regulation of translation initiation. For the majority of eukaryotic mRNAs, recruitment is dependent upon the activity of the translation initiation factor eIF4E. eIF4E binds to the 5'-m^7^GTP-cap structure of mRNAs and to the initiation factor eIF4G (reviewed [1-3]). Through the interaction of the eIF4E:eIF4G complex with ribosome bound factor eIF3, the 40 S ribosomal subunit is positioned at the 5'-end of the mRNA. Subsequently, the 40 S ribosomal subunit scans the mRNA (5'-3') for the translational start codon prior to 60 S binding and formation of the first peptide bond.

The crystal structures of eIF4Es from *Mus musculus *and *Homo sapiens *and the solution structure of eIF4E from *Saccharomyces cerevisiae*, in each case bound to cap-analogues, show that each consists of an eight-stranded β-sheet supported by three α-helices forming the palm and back of a 'cupped' hand [4-6]. Two conserved aromatic Trp residues (Trp-56 and Trp-102 for *H. sapiens *eIF4E) grasp the aromatic guanine residue of the cap-structure through 'π'-bond interactions [4,5]. Similar interactions of aromatic amino acid residues with the guanine nucleotide of cap-analogues are seen in the structures of other cap-binding proteins which appear to have evolved independently such as the vaccinia virus 2'-O-methyltransferase, VP39, and of the nuclear cap-binding protein subunit, CBP20, suggesting a common evolutionary theme for methylguanosine/nucleotide-interaction [7]. Hydrogen bonds to the guanine base from a Glu residue (Glu-103 of *H. sapiens *eIF4E) and the adjacent peptide bond stabilize the interaction of the cap-analogue to the protein. A third Trp residue (Trp-166 of *H. sapiens *eIF4E) interacts with the N^7^-methyl moiety of the cap-structure. Sequence comparisons of mammalian eIF4E with eIF4Es from plants and *S. cerevisiae*, coupled with deletion analyses of eIF4Es from *S. cerevisiae *and *Danio rerio*, suggest that the N- and C-termini of eIF4E are dispensable for translation and that the core of eIF4E represented by ~170 amino acids (from His-37 to His-200 in *H. sapiens *eIF4E) is sufficient for binding to the cap-structure and to eIF4G and 4E-BPs [8,9]. However, the N- and C-termini may be involved in the regulation of eIF4E-activity [10,11] or affect the stability of the protein.

eIF4E-activity is regulated by the actions of eIF4E-binding proteins or 4E-BPs which share sequence similarity with the eIF4E-binding domain within the N-terminal region of eIF4G (reviewed [12]). 4E-BPs act as competitive inhibitors of eIF4E-eIF4G interaction [13-15]. Crystal structures of mouse eIF4E bound to 4E-BPs and fragments of eIF4G show that both proteins interact with eIF4E via a common mechanism involving a sequence with the consensus YxxxxLΦ (where Φ is a hydrophobic residue) [16]. Hyper-phosphorylation of 4E-BPs occurs following stimulation of the Akt/FRAP/TOR signal transduction pathway and results in a reduced affinity for eIF4E [17-19].

Studies of *M. musculus *eIF4E bound to either a fragment of eIF4G or 4E-BP1 have revealed that His-37, Pro-38, Val-69, Trp-73, Leu-131, Glu-132, and Leu-135 (numbers for *H. sapiens *eIF4E) interact with the eIF4E-binding regions within eIF4G and 4E-BPs [16]. Val-69 and Trp-73 are within a conserved sequence of the consensus (S/T)V(e/d)(e/d)FW (where the acidic residues are not completely conserved). Substitution of a non-aromatic amino acid for Trp-73 has been shown to disrupt the ability of eIF4E to interact with eIF4G and 4E-BPs [20,21]. Substitution of a Gly residue in place of Val-69 creates an eIF4E variant that binds still binds 4E-BP1 but has a reduced capacity to interact with both eIF4G and 4E-BP2 [21].

eIF4E is ubiquitously expressed and is generally isolated from cell extracts using m^7^GTP-affinity matrices. Use of such matrices led to the conclusion that in mammalian cells, eIF4E was represented by a single polypeptide of ~25 kDa. Similar chromatographic resolution of proteins from plant cell extracts suggested that plants differ from mammalian cells in that they contain two different but related proteins termed plant eIF4E and eIF(iso)4E, or p26 and p28 (in reference to their apparent molecular weights as judged by SDS-PAGE) [22,23]. A gene encoding eIF4E from *S. cerevisiae *was isolated and shown by southern analyses and gene disruption studies to be the sole eIF4E gene in that organism [24], a conclusion confirmed by the availability of the sequence of the complete genome. *S. cerevisiae *lacking a functional eIF4E-gene can be rescued by exogenous expression of mammalian eIF4E showing that *S. cerevisiae *and mammalian eIF4Es are structurally and functionally comparable [24]. Overall, these findings suggested that, with the exception of plants, organisms contain a single gene that encodes eIF4E.

Growing evidence from genome/EST sequencing projects has revealed that many organisms contain multiple genes encoding proteins that have sequence similarity to recognized, or prototypical, eIF4E proteins such as mammalian eIF4E (reviewed in [25-27]). Consequently, the translation factor eIF4E and its relatives comprise a family of structurally related proteins within a particular organism. To distinguish the recognized vertebrate eIF4E from its relatives, vertebrate eIF4E has since been renamed eIF4E-1 [28] (or eIF4E-1A [9]). The functions of the eIF4E-related proteins are not yet understood. Some may act as translation factors and stimulate global mRNA recruitment, or specifically the recruitment of a subset of mRNAs [29,30]. Others may possess only partial activities when compared to prototypical eIF4Es [26,31] and thus act as inhibitors of mRNA recruitment. Lack of detection of these eIF4E related proteins in fractions derived from cell extracts resolved by m^7^GTP-affinity chromatography may reflect a variety of underlying causes. The proteins may be expressed ordinarily at low levels, at specific developmental times, or in restricted and untested tissues [9,26,30,32]. Alternatively, they may be unresolved from eIF4E-1 in fractionation by standard polyacrylamide gel electrophoresis. Conversely, they may fail to interact stably with [26], or recognize structures that differ from, the m^7^GTP-cap-structure [28,33,34] preventing isolation using standard m^7^GTP-affinity resins.

At the time of writing, BLAST search of the NCBI GenBank NR database using the amino acid sequence of *M. musculus *eIF4E-1 as a probe recovers <100 unique cDNA sequences of eIF4E-family members with expected values below 14. Only some of these are recognized as eIF4E-family members in the GenBank database. Additional eIF4E-family member sequences can be uncovered from genomic sequences, although these predicted sequences are subject to errors arising from less than adequate predictions of intron/exon boundaries. Through mining of the GenBank dbEST database and assembling sequences of overlapping cDNA fragments to derive consensus cDNA sequences, as well as performing reiterative searches, we have been able to extend the number of identified complete or partial cDNA sequences encoding eIF4E-family members to 379 (derived from 204 taxonomic species). A further 32 eIF4E-family members from 26 additional species can be predicted from the genomic sequences of organisms known to lack or contain few introns in genes transcribed from RNA polymerase II promoters. The sequences of identified eIF4E-family members have been deposited in an internet-accessible database designed for sequence analyses of eIF4E-family members [35]. Analyses of the sequences suggest that the eIF4E-structure has been duplicated numerous times during evolution producing new forms of the protein that may serve other tasks or regulate the activities of the prototypical translation factor.

## Results and discussion

### Definition of the amino acid core of an eIF4E-family member

**Figure 1:**
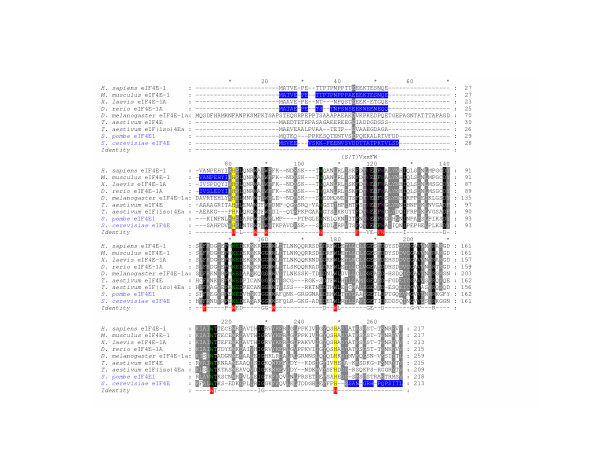
**An alignment of the amino acid sequences of selected established eIF4E-family members**. An alignment of the complete amino acid sequences of *H. sapiens *eIF4E-1, *M. musculus *eIF4E-1, *X. laevis *eIF4E-1A, *D. rerio *eIF4E-1A, *D. melanogaster *eIF4E-1a, *T. aestivum *eIF4E and eIF(iso)4E, *S. pombe *eIF4E1, and *S. cerevisiae *eIF4E. eIF4E-family members with names in blue indicate that the sequence was estimated or verified using genomic sequence data. A sequence of identity is shown with aromatic residues boxed in red. Black and grey shading: conserved amino acids identical in all or similar in greater than 75 % of the sequences shown, respectively. Yellow shading: His-residues that border the conserved core region of an eIF4E-family member. Blue shading: regions of the respective eIF4E-family member that have been shown to be dispensable for eIF4E-function *in vitro*. Residues in green: positions of residues equivalent to Trp-56, Trp-102, Glu-103 and Trp-166 of *H. sapiens *and *M. musculus *eIF4E-1 that directly interact with the cap-structure. Residues in purple: identity with respect to residues Val-69 and Trp-73 of *M. musculus *eIF4E-1 that interact with eIF4G and 4E-BPs and are found within a region of eIF4E-family members possessing the concensus (S/T)VxxFW (as indicated). Numbers to the right of the sequences indicate the positions of residues from the N-terminal Met.

Alignment of the complete amino acid sequences of the *bonafide *translation initiation factors *M. musculus *eIF4E-1, *D. rerio *eIF4E-1A, *D. melanogaster *eIF4E-1, *T. aestivum *eIF4E and eIF(iso)4E, *Schizosaccharomyces pombe *eIF4E1, and *S. cerevisiae *eIF4E, suggests the presence of an evolutionarily conserved "core" region (Figure 1). The region stretches approximately 160–170 residues from His-37 to His-200 of *H. sapiens *and *M. musculus *eIF4E-1. Evidence supporting the designation of this region as a functional core comes from deletion analyses [8,9]. The variant of *D. rerio *eIF4E-1A, eIF4E-1A(Δ1–33), lacking sequences N-terminal to within one amino acid of the conserved core region, is able to rescue the growth of *S. cerevisiae *lacking a functional *eIF4E*-gene [9]. Also, the variant of *M. musculus *eIF4E-1, eIF4E-1(Δ1–35), lacking N-terminal residues up to Lys-36, can bind both the cap-structure, the N-terminal fragment of eIF4G, and 4E-BPs *in vitro *[16]. Similarly, the *S. cerevisiae *eIF4E variants eIF4E(Δ1–29/Δ207–213) and eIF4E(Δ201–213) remain active in their ability to rescue the growth of *S. cerevisiae *lacking a functional *eIF4E*-gene and to bind the cap-structure *in vitro *[8]. As evident in Figure 1, regions N- and C-terminal to the defined core are not conserved in all *bonafide *eIF4E proteins. These regions may be involved in the regulation of eIF4E-activity [10,11] or may affect the stability of the protein. The N-terminal residues of *S. cerevisiae *eIF4E interact have been found to interact with eIF4G and stabilize the interaction [10]. The C-terminal residues of *bonafide *eIF4Es from Metazoa and *S. pombe*, but not of Viridiplantae possess a phosphorylatable Ser residue (Ser-209 of H. sapiens eIF4E-1) which in mammalian eIF4E has been shown to change the binding affinity of eIF4E for the cap structure [11,36]. The consensus sequence of the conserved core region suggests characteristics for a protein to be defined as a member of the eIF4E-family. Aromatic residues Trp, Phe, and His show a distinctive pattern across from N- to C-terminus summarized by H(x_5_)W(x_2_)W(x_8–12_)W(x_9_)F(x_5_)FW(x_20_)F(x_7_)W(x_10_)W(x_9–12_)W(x_34–35_)W(x_32–34_)H.

### Acquisition of nucleotide sequences encoding putative eIF4E-family members

In order to obtain nucleotide coding sequences representing an accurate description of the repertoire of functional genes encoding eIF4E-family members within an organism it was decided that some evidence should support the expression of the eIF4E-family member for which sequence is obtained. Consequently, for the most part, sequences of expressed sequence tags (ESTs) were acquired. In general, direct use of genome sequences was avoided due to the possibility of including pseudogenes and the possible inaccuracy with which intron/exon boundaries can be predicted. However, where sufficient EST data to verify nucleotide sequences encoding the core region of an eIF4E-family member was absent, it was considered reasonable to use genome sequences for confirmation. Furthermore, the use of genome sequences was considered valid for organisms whose genomes are known to lack, or contain few, introns in genes transcribed by RNA polymerase II such as some Protista and yeasts. In such cases, genome sequences were used only if sequences indicated that indeed no introns were present in the gene representing an eIF4E-family member and that expressed cDNAs could be identified in the same, or closely related organisms.

Expressed nucleotide sequences encoding putative eIF4E-family members were acquired from GenBank NR and dbEST databases by using the nucleotide and amino acid sequences encoding *M. musculus *eIF4E-1, eIF4E-2, and eIF4E-3, *T. aestivum *eIF4E and eIF(iso)4E, *A. thaliana *nCBP, *C. elegans *IFE-1, 2, 3, 4, and 5, and *S. cerevisiae *eIF4E as probes for BLAST searches. Sequences encoding putative eIF4E-family members were easily identified by comparison of computed translations and the consensus pattern for the conserved core region described above. The retrieved eIF4E-related sequences were used to re-probe the databanks to retrieve further sequences of overlapping cDNA fragments from the same species or to obtain sequences from additional species. The process of iteration was continued to obtain sequences encoding more eIF4E-family members. Genomic sequences from organisms known to contain few introns in genes transcribed from RNA pol II promoters were also probed in a similar manner. In all 2,383 nucleotide sequences were collected representing nucleotide sequences from 230 species. The statistics of sequence acquisitions and alignments are presented in Table 1 and 2.

**Table 1:** Overall statistics of the dataset of nucleotide sequences encoding eIF4E-family members

**Databank from which sequences were acquired**	**Number of sequences**	**Number of species**	**Number of eIF4E-family members**
GenBank^1 ^dbEST^2^	2,237	191	356
GenBank NR^3^	80	32	59
GenBank Genomic^4^	53	33	42
Genome Projects^5^	13	1	1

Total^6^	2,383	230	411

^1^GenBank: the NIH genetic sequence database. ^2^GenBank database of EST sequences (dbEST). ^3^mRNA sequences derived from the GenBank database (excluding those within the dbEST databank and any predicted from genomic sequences). ^4^Predicted mRNA sequences derived from genomic sequences deposited in the GenBank database. ^5^Sequences predicted from genomic DNA assemblies not yet submitted to GenBank. ^6^The total dataset (accessible via the internet at "The eIF4E-family member database" [35]).

**Table 2:** Identification and verification of nucleotide sequences encoding eIF4E-family members

**Region within the coding sequences of an eIF4E-family member**	**Number of eIF4E-family members**	
	**Identified**	**Verified**^**4**^
	
Start Codon^1^	278	155
Stop Codon^2^	259	149
Complete coding sequence	200	105
Sequence encoding the entire core region^3^	243	120 (142 >90%)

Total dataset (any region identified)	411	NA

^1^Initiation of translation was assumed to occur at ATG codons. ^2^The first of any of UGA, UAG, and UAA codons 3' and in frame with core regions were assumed to be translational stop codons. ^3^Core regions defined as regions including codons representing equivalents of *H. sapiens *eIF4E-1 residues His-37 to His-200. ^4^Number of eIF4E-family members for which the sequence or region indicated could be verified by two or more sequence reads.

Nucleotide sequences encoding an eIF4E-family member from a particular species were aligned to produce complete or partial consensus cDNA sequences. In many cases the initiation and/or stop codons could not be accurately identified because of a lack of overlapping clones to remove sequence errors or a lack of clones representing full length cDNA products. However, sufficient sequence information was usually available to identify the complete core regions. Analyses of consensus sequences encoding 220 representative eIF4E-family members from 118 species of eukaryotes are presented.

### The eIF4E-family of proteins

**Figure 2:**
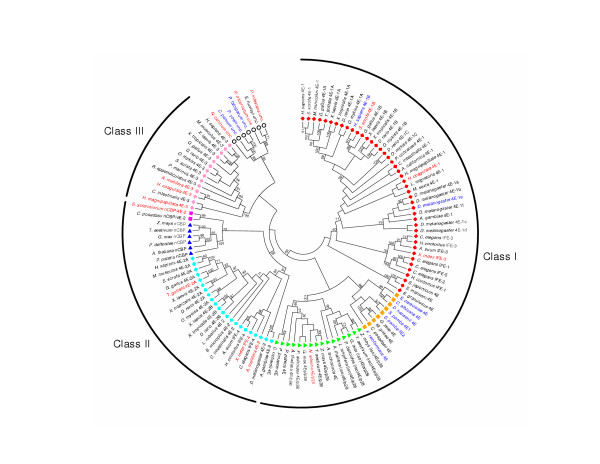
**A radial cladogram describing the overall relationship of selected eIF4E-family members from multiple species**. The topology of a neighbor-joining tree visualized in radial format derived from an alignment of nucleotide sequences representing the conserved core regions of the indicated eIF4E-family members. The full names of the species represented and the accession numbers for cDNA sequences used to derive consensus core sequences can be found within supplementary data to this publication. Alignments of cDNA sequences to derive consensus core sequences can be obtained and verified at the "**eIF4E-family member database**" [35]. eIF4E-family member names in black or red indicate whether or not the complete sequence of the conserved core region of the member could be predicted from consensus cDNA sequence data, respectively. eIF4E-family member names in blue indicate that genomic sequence data was used to either verify or determine the nucleotide sequence representing the core region of the member. The shape of a 'leaf' indicates the taxonomic kingdom from which the species containing the eIF4E-family member derives: Metazoa (diamonds); Fungi (squares); Viridiplantae (triangles); and Protista (circles); respectively. The color of a 'leaf' indicates the sub-group of the eIF4E-family member: metazoan eIF4E-1 and IFE-3-like (red); fungal eIF4E-like (gold); plant eIF4E and eIF(iso)4E-like (green); metazoan eIF4E-2-like (cyan); plant nCBP-like (blue); fungal nCBP/eIF4E-2-like (purple); metazoan eIF4E-3-like (pink); atypical eIF4E-family members from some protists(white). eIF4E-family members within structural classes Class I, Class II, and Class III are indicated. Bootstrap values of greater than 60 % derived from 50,000 tests are shown.

**Figure 3:**
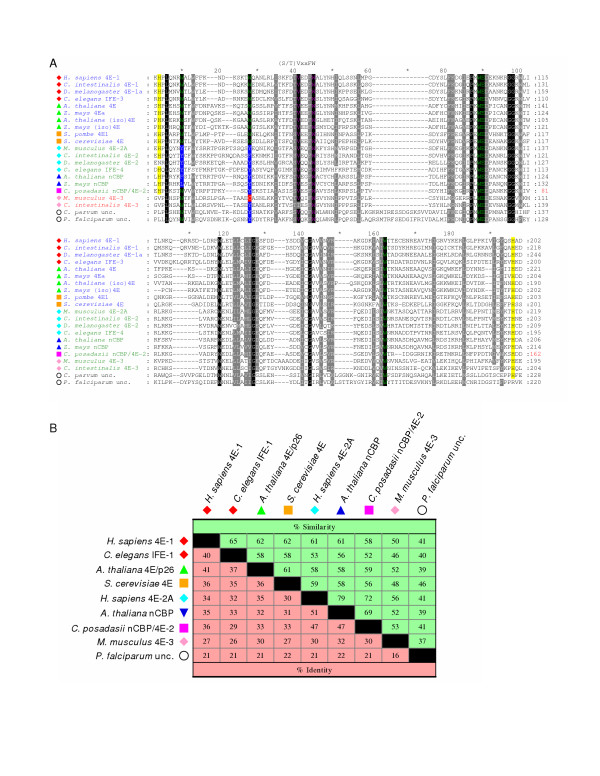
**Comparison of the conserved cores of eIF4E-family members from different taxonomic sub-groups**. **A. **An alignment of amino acid sequences representing the conserved core regions of the indicated eIF4E-family members. Sequence names are highlighted to indicate structural class: Class I in blue; Class II in green; and Class III in red. Atypical eIF4E-family members that could not be accurately classified based on similarity to other structural class members are shown with sequences names in black. Symbols to the left indicate the taxonomic sub-group of the eIF-4E-family member (as described in the legend to Figure 2). Residues highlighted within the amino acid alignment represent: identity with respect to residues Trp-43, Trp-56, Trp-102, Glu-103, and Trp-166 within *H. sapiens *eIF4E-1 (green); identity within the conserved (S/T)VxxFW consensus region containing amino acids equivalent to Val-69 and Trp-73 of *H. sapiens *eIF4E-1(purple); identity with His-residues equivalent to those that border the core region of *H. sapiens *eIF4E-1 (shaded in yellow). Variations at residues equivalent to Trp-43 and Trp-56 of *H. sapiens *eIF4E-1 are indicated as follows: Tyr/Phe-shaded in blue with white text; Cys-shaded in red with white text. Residues shaded in black or grey within the alignment indicate amino acids that are identical in all sequences or similar in greater than 85% of the sequences, respectively. Numbers to the right of the alignment represent distances of amino acids with respect to the predicted N-terminal Met residue. **B. **Identities and similarities (based on a PAM 250 matrix [58]) between the amino acid sequences representing the core regions of selected eIF4E-family members from each of the eight sub-groups.

Dissection of the dataset by taxonomic criteria is presented in Table 3. The majority of acquired sequences represented eIF4E-family members from Metazoa (Animalia), Viridiplantae and Fungi. Comparison of the number of eIF4E-family members defined within a particular taxonomic division and the number of species the members represented suggests that many organisms contain two or more eIF4E-family members. A radial view of a cladogram derived from an alignment of the nucleotide sequence representing conserved core regions of selected eIF4E-family members from >100 species of Viridiplantae, Metazoa, Fungi, and Protista is presented in Figure 2. Based on branching and clustering, the eIF4E-family can be separated taxonomically into eight sub-groups consisting of 1) the metazoan eIF4E-1 and nematode IFE-3-like; 2) plant eIF4E and eIF(iso)4E-like; 3) fungal eIF4E-1-like; 4) metazoan eIF4E-2-like; 5) plant nCBP-like; 6) fungal nCBP/eIF4E-2-like; 7) metazoan eIF4E-3-like; and 8) a set of atypical eIF4E-family members found in certain protists. Additional eIF4E-family members have been found that fail to fall into any of these subgroups. These are not included in the analysis in Figure 2, but some are discussed in a later section. An alignment of representative members from the eight sub-groups are presented in Figure 3A and comparisons of identities and similarities between the amino acid sequences representing the core regions of selected eIF4E-family members from each of the eight sub-groups are presented in Figure 3B. Variations at residues equivalent to *H. sapiens *Trp-56 and at Trp-43 of eIF4E-1 (shown in green in Figure 3A) provide a convenient means to categorize the non-protist eIF4E-family members into three classes on a structural basis. With the exception of Trp-56, residues of *H. sapiens *and *M. musculus *eIF4E-1 that have been shown to directly interact with the mRNA-cap structure (Trp-56, Trp-102, Glu-103, and Trp-166) are identical in all classified eIF4E-family members for which corresponding residues could be identified.

**Table 3:** Dissection of dataset with respect to taxonomic divisions

**Taxonomic division**	**No. of eIF4E-family members identified**^**1**^	**No. of species represented**^**2**^	**eIF4E-family members/species**
Metazoa (Animalia)	186 (186)	89 (89)	2.09
Viridiplantae	155 (155)	83 (83)	1.87
Fungi	42 (18)	36 (16)	1.17
Protista	28 (20)	22 (16)	1.27

Total	411 (379)	230 (204)	1.78

^1^The number of eIF4E-family members for which nucleotide coding sequences were identified. In parentheses: the number of eIF4E-family members identified excluding those based only on genomic sequences. ^2^The number of species (organisms) represented for which nucleotide sequences encoding an eIF4E-family member could be identified. In parentheses: the number of species represented excluding those for which genomic sequences were the only source for identification of an eIF4E-family member.

### Class I eIF4E-family members

**Figure 4:**
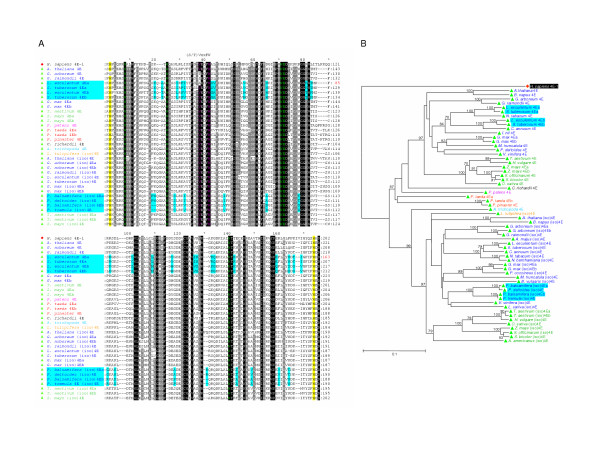
**Comparison of the conserved core regions of selected Class I eIF4E-family members from Viridiplantae**. **A. **An alignment of the amino acid sequences representing the 'core' regions of Class I eIF4E-family members from the indicated species of Viridiplantae and of eIF4E-1 from *H. sapiens*. Amino acid residues within the alignment are highlighted as described in the legend to Figure 3A with the exception that residues shaded in grey indicate similar amino acids in more than 90% of the sequences shown. Numbers to the right of the alignment represent distances of amino acids with respect to the N-terminal Met residue (black) or, for eIF4E-family members for which the N-terminal Met could not be predicted, from the first residue shown (red). **B. **A phylogram constructed by neighbor-joining derived from alignments of nucleotide sequences representing the core regions of the indicated Class I-family members. Bootstrap values greater than 70% derived from 50,000 tests are shown to indicate supported nodes. For **A **and **B**: names of eIF4E-family members are highlighted to indicate taxonomic divisions: Eudicotyledons (blue), Liliopsida (green), Bryopsida (purple), Coniferopsida (red), Stem Magnoliophyta (cyan), Magnoliids (orange), Chlorophyceae (black), Mammalia (white on black). Names of family members and residues shaded in cyan indicate evidence that a gene-duplication occurred prior to speciation.

**Figure 5:**
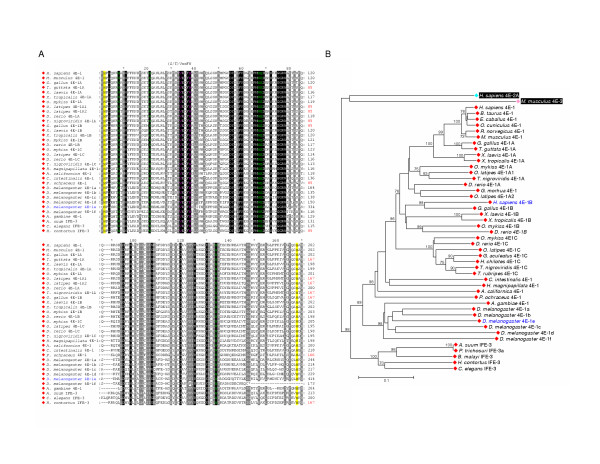
**Comparison of the conserved core regions of selected Class I eIF4E-family members from Metazoa**. **A. **An alignment of the amino acid sequences representing the 'core' regions of Class I eIF4E-family members from the indicated species of Metazoa. Amino acid residues within the alignment are highlighted as described in the legend to Figure 3A with the exception that residues shaded in grey indicate similar amino acids in more than 95% of the sequences shown. Numbers to the right of the alignment represent distances of amino acids with respect to the N-terminal Met residue (black) or, for eIF4E-family members for which the N-terminal Met could not be predicted, from the first residue shown (red). **B. **A phylogram constructed by neighbor-joining derived from alignments of nucleotide sequences representing the core regions of the indicated Class I-family members. Bootstrap values greater than 70% derived from 50,000 tests are shown to indicate supported nodes. For **A **and **B**: names of eIF4E-family members highlighted in blue indicate that genomic sequence from the indicated species was employed to verify and predict the amino acid sequence of the eIF4E-family member.

**Figure 6:**
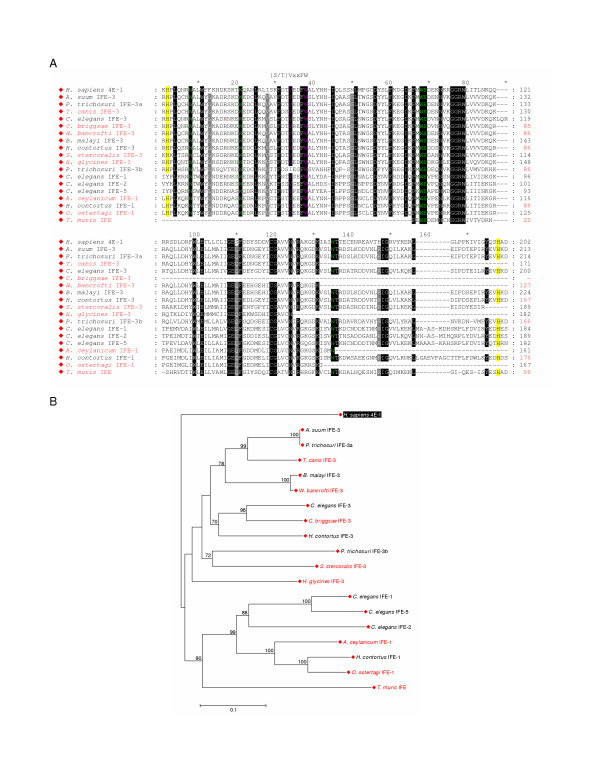
**Comparison of the conserved core regions of Class I eIF4E-family members from species of Nematoda**. **A. **An alignment of amino acid sequences representing the conserved core regions of Class I eIF4E-family members from the species of Nematoda indicated and of *H. sapiens *eIF4E-1. Amino acid residues within the alignment are highlighted as described in the legend to Figure 3A with the following exceptions: residues shaded in black indicate amino acids identical in all eIF4E-family members with respect to regions that could be predicted; residues shaded in grey indicate amino acids identical in all eIF4E-family members from nematoda with respect to regions that could be predicted that differ from equivalent residues in *H. sapiens *eIF4E-1. Numbers to the right of the alignment represent distances of amino acids with respect to the N-terminal Met residue (black) or, for eIF4E-family members for which the N-terminal Met could not be predicted, from the first residue shown (red). **B. **A phylogram constructed by neighbor-joining derived from an alignment of nucleotide sequences representing the conserved core regions of the eIF4E-family members indicated. Bootstrap values greater than 70% derived from 50,000 tests are shown to indicate supported nodes. For **A **and **B**: names of eIF4E-family members in red indicate that only a portion of the conserved core region could be predicted.

Structural Class I of the eIF4E-family members include of members of sub-groups 1, 2, and 3 (Figure 2). cDNAs encoding members of structural Class I can be identified in species from Viridiplantae, Metazoa, and Fungi. As judged from completed genomes, many protists also encode Class I-like family members (data not shown). The Class I family includes orthologues of the prototypical eIF4Es described for *H. sapiens *(eIF4E-1), *M. musculus *(eIF4E-1), *T. aestivum *(eIF4E and eIF(iso)4Es), and *S. cerevisiae *(eIF4E) (reviewed [1,3]). Comparisons of the amino acid sequences representing the core regions of selected members from each of the sub-groups 1, 2, and 3, reveal that they share ~35–40 % identity and ~60–65% similarity with one another (Figure 3B). Alignments and the relationships of selected representative Class I eIF4E-family members are shown in Figures 4, 5, and 6. All identified members of structural Class I (subgroups 1, 2 and 3) possess Trp residues at the equivalents of Trp-43 and Trp-56 of *H. sapiens *eIF4E-1 and include metazoan eIF4E-1s plant eIF4E(p26) and eIF(iso)4E(p28), and fungal eIF4E. Examination of sequences of members from each of the sub-groups reveals evidence of gene duplications.

Evidence supporting the presence of two distinct Class I sub-group 2 eIF4E-family members represented by viridiplantae eIF4E and eIF(iso)4E, can be found in species from the viridiplantae classes Liliopsida and Eudicotyledons. Sequences representing either one of eIF4E or eIF(iso)4E can be identified in other viridiplantae classes suggesting that the genes arose from an earlier duplication event. The two forms are closely related in sequence (Figure 4A and 4B) and each possesses all the activities attributed to mammalian eIF4E-1 *in vitro *[23,37]. However, expression of *A. thaliana *eIF(iso)4E in *S. cerevisiae *lacking a functional endogenous eIF4E-gene results in slower growth relative to similar expression of *A. thaliana *eIF4E [38]. In addition, levels of expression of *A. thaliana *eIF4E and eIF(iso)4E differ in various *A. thaliana *tissues; eIF4E is expressed ubiquitously (with the exception of tissues in the zone of specialization of the root); eIF(iso)4E is expressed more abundantly in developing tissues [38]. Subtle differences in their relative activities can be inferred from the requirements for each in potyvirus infected cells. Both plant eIF4E and eIF(iso)4E bind to potyviral genome-linked proteins (Vpgs) [39]. However, strains of *A. thaliana *lacking eIF(iso)4E, or of *P. sativum *carrying variants of eIF4E which lack cap-binding ability lose susceptibility to potyvirus infection [40,41].

In certain plant species from Eudicotyledons, Liliopsida and Coniferospida, multiple forms of eIF4E and eIF(iso)4E can be found. Instances of apparent gene duplication can be seen in species such as *Zea mays *and *Triticum aestivum *with respect to eIF4E (p26) and eIF(iso)4E (p28). However, there is no evidence to support the hypothesis that these duplications occurred prior to speciation. In contrast, evidence suggesting gene duplication prior to speciation can be found (compare eIF4E-family member names and residues shaded in cyan in Figure 4A and 4B) in the Solanaceae, in which *Lycopersicon esculentum *(tomato) and *Solanum tuberosum *(potato) both have two forms of eIF4E(p26) (A and B), and in Salicaceae which have two forms of eIF(iso)4E(p28) (A and B).

Evidence for gene duplication of Class I eIF4E-family member genes eIF4E-1 can also be found in Metazoa with respect to Chordata, Insecta and Nematoda. Three eIF4E-1 sub-family members can be identified from the zebrafish *Danio rerio*, termed eIF4E-1A, eIF4E-1B [9] and eIF4E-1C (Figure 5A and 5B). Orthologues of the gene encoding eIF4E-1B, but not that of eIF4E-1C, can be found in almost all species above Actinoptergyii for which sequence has been acquired. Both eIF4E-1A and eIF4E-1B possess similar levels of identity when compared to mammalian eIF4E-1s and possess all known residues required for interaction with the cap-structure, eIF4G, and 4E-BPs. eIF4E-1A, like mammalian eIF4E-1, is expressed ubiquitously and can restore the growth of *S. cerevisiae *lacking a functional *eIF4E*-gene [9]. It can bind to the cap-structure, eIF4G, and 4E-BP *in vitro *[9]. Conversely, eIF4E-1B is expressed only during early embryogenesis and in the gonads and muscles of adult fish, and is unable to complement yeast lacking eIF4E. Furthermore, eIF4E-1B is unable to bind to the cap-structure, eIF4G, or 4E-BP *in vitro*.

*Drosophila melanogaster *has a total of six genes, all of which are expressed, encoding Class I eIF4E-family members eIF4E-1a-f (also termed eIF4E-1, 4, 5, 3, 7, and 6, respectively [42]). There is also evidence of a seventh Class I eIF4E-family member (termed eIF4E-2 in [42]) that arises from alternate splicing of primary transcripts and shares the same core sequence as eIF4E-1a. Four of the genes share exon/intron structure in their carboxy-terminal regions and form a cluster in the genome. All Class I eIF4Es from *D. melanogaster *bind to cap-analogue. Furthermore, all of them, except eIF4E-1f (eIF4E-6 in [42]), which has a truncated carboxy-terminal domain, are able to interact with *D. melanogaster *eIF4G or 4E-BP. The expression of each has been shown to vary throughout the life cycle of the fly. Examination of both expressed sequences and partial or complete genome sequences of insect species has not revealed a similar repertoire of Class I eIF4E-family members outside the genus of *Drosophila *(data not shown).

An alignment of Class I eIF4E-family members from nematodes is presented in Figure 6A. Although only a single Class I eIF4E orthologue can be found in *Ascaris suum*, many nematodes express more than one Class I family member (Figure 6B). Four of the five *C. elegans *eIF4E-family members (termed IFEs for initiation factor of *elegans*), are Class I members. With respect to activities, IFE-3 corresponds to mammalian eIF4E-1 and binds only to mono-methylated cap-structures. However, in nematodes, a proportion of the mRNAs possesses a tri-methyl-cap arising from the post-transcriptional addition of a tri-methyl-cap containing spliced leader RNA (SLRNA) to the 5' end of a transcribed mRNA [43,44]. The translation of such trans-spliced mRNAs in *C. elegans *is thought to be mediated by IFE-1, 2, and 5 since they, unlike IFE-3, interact with both mono- and tri-methylated cap-structures [28,33]. IFE-1, 2, and 5 possess more similarity to IFE-3 in sequence than to Class I family members from other phyla of Metazoa suggesting they arose from gene-duplications of a progenitor IFE-3 (Figure 2 and 6B). Evidence in support of this hypothesis comes from recent studies of the only identified IFE-3-like protein from the nematode *Ascaris suum. A. suum *IFE-3 (also termed eIF4E-3) can bind and stimulate the translation of mRNAs possessing mono- or tri-methylated cap structures [45]* in vitro*. Furthermore, identified sequences from some nematodes, such as the parasitic *Haemanchus contortus *suggest that they express single form of eIF4E similar to IFE-3 and a single form related to IFE-1, -2 or -5. No direct relatives of IFE-1, -2 and -5-like proteins have been found in any taxonomic group other than Nematoda.

Multiple Class I eIF4E-family members can also be found in the fission yeast *Schizosaccharomyces pombe. S. pombe *expresses two eIF4E-family members that share 52 % identity termed eIF4E1 and eIF4E2 [46]. Unlike eIF4E1, expression of eIF4E2 is not essential. However, levels of eIF4E1 and eIF4E2 vary with growth temperature and at higher temperatures eIF4E2 is more abundant than eIF4E1. Both eIF4E1 and eIF4E2 can bind the cap-structure with similar affinity *in vitro *but eIF4E1 has a 100-fold greater affinity for *S. pombe *eIF4G than eIF4E2 despite the fact that both share all known amino acids required for interaction with eIF4G. No evidence supporting the expression of two genes directly related to those for *S. pombe *eIF4E1 and eIF4E2 has been found.

### Class II eIF4E-family members

**Figure 7:**
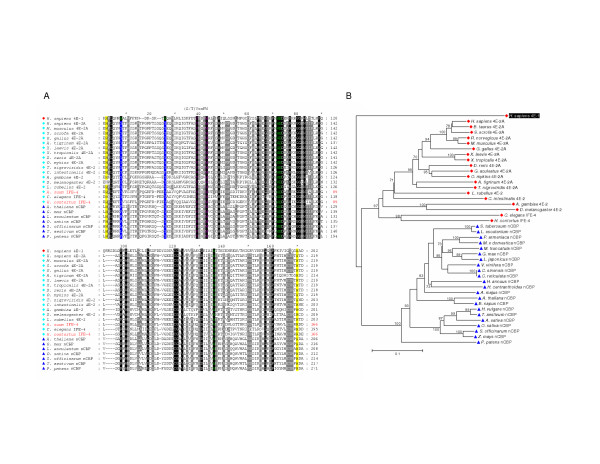
**Comparison of the conserved core regions of selected Class II eIF4E-family members**. **A. **An alignment of amino acid sequences representing the conserved core regions of the Class II eIF4E-family members from the taxonomic species indicated and of *H. sapiens *eIF4E-1. Amino acid residues within the alignment are highlighted as described in the legend to Figure 3A with the exception that residues shaded in grey indicate identical amino acids in greater than 84% of the sequences shown. Numbers to the right of the alignment represent distances of amino acids with respect to the N-terminal Met residue (black) or, for eIF4E-family members for which the N-terminal Met could not be predicted, from the first residue shown (red). **B. **A phylogram constructed by neighbor-joining derived from an alignment of nucleotide sequences representing the conserved core regions of the eIF4E-family members indicated. Bootstrap values greater than 70% derived from 50,000 tests are shown to indicate supported nodes. For **A **and **B**: names of eIF4E-family members in red indicate that only a portion of the conserved core region could be predicted.

Structural Class II members include eIF4E-2-family members from Metazoa (sub-group 4) and nCBP-family members from Viridiplantae (sub-group 5). Class II eIF4E-family members can also be recognized in pathogenic fungi from the sub-phylum Pezizomycotina, including *Coccidioides posadasii *and *Sclerotinia schleroiorum *(sub-group 6, Figure 2) but are absent in the model ascomycetes, *S. cerevisiae *and *S. pombe*. An alignment and the relationships of representative Class II eIF4E-family members is presented in Figure 7A and 7B, respectively. Comparisons of amino acid sequences representing the core regions of selected sub-group 4, 5, and 6 eIF4E-family members show that they share approximately 50 % identity and 70–80 % similarity with one another (Figure 3B). The members also posses ~30–35 % identity and 60–65 % similarity to Class I eIF4E-family members of sub-groups 1, 2, and 3. Like Class I eIF4E-family members, Class II eIF4Es also share a structural core of approximately ~160–170 amino acids. All identified members of this class differ from Class I eIF4E-family members in that they possess a hydrophobic residue such as Tyr, Phe, or Leu, but not Trp, in the position equivalent to Trp-56 of *H. sapiens *eIF4E-1. All identified Class II members from Metazoa and Viridiplantae also contain a substitution at the position equivalent to Trp-43 of *H. sapiens *eIF4E-1. However, this substitution has not so far been seen in the few Class II members identified in Fungi.

Studies have shown the Class II eIF4E-related proteins eIF4E-2A (*H. sapiens *and *M. musculus*; also termed eIF4E-2, 4EHP or 4E-LP), IFE-4, (*C. elegans*), *D. melanogaster *eIF4E-2 (eIF4E-8 in [42]), and nCBP (*A. thaliana*), like the mammalian translation factor eIF4E-1, all bind the m^7^GTP-cap structure [26,29,31,42]. nCBP from *A. thaliana *differs from *H. sapiens and Mus musculus *eIF4E-2A and *D. melanogaster *eIF4E-2 in that *A. thaliana *nCBP can interact with eIF4G and participate in productive translation [29], whereas *H. sapiens *and *M. musculus *eIF4E-2A and *D. melanogaster *eIF4E-2 cannot [26,42]. Consistent with this observation, *M. musculus *eIF4E-2A and *D. melanogaster *eIF4E-2 cannot substitute for *S. cerevisiae *eIF4E in a strain lacking a functional *eIF4E*-gene [26,42]. Recent studies have shown that *H. sapiens *and *M. musculus *eIF4E-2A can interact with 4E-BPs but to a lesser degree than mammalian eIF4E-1 [26,47]. Although mammalian eIF4E-2A mRNA appears to be expressed in all tissues, the levels of *M. musculus *eIF4E-2A protein are ~10-fold lower than eIF4E-1 [31].

Given that metazoan eIF4E-2 cannot itself partake in protein synthesis due to its inability to interact with eIF4G, growing evidence suggests a regulatory role for metazoan eIF4E-2 family members. Like mammalian eIF4E-2A, *D. melanogaster *eIF4E-2 (eIF4E-8 in [42]) appears to expressed at much lower levels than the major Class I form, eIF4E-1a (eIF4E-1 in [42]), although it is present at all stages of the life cycle. Mutants of *D. melanogaster *that express a markedly reduced level of eIF4E-2 show defects in anterior-posterior axis formation during early embryogenesis [48]. Development of the anterior-posterior axis in *D. melanogaster *embryos is dependent on the distribution of the maternal effect genes which include *bicoid *and *caudal*. In the oocyte, caudal mRNA is evenly distributed, whereas bicoid mRNA is restricted to the anterior of the cell. Translation of bicoid mRNA is activated upon fertilization resulting in a gradient of bicoid protein decreasing toward the posterior of the embryo. Through interaction of bicoid with a region within the 3' UTR of caudal mRNA, the translation of caudal mRNA is inhibited resulting in an opposing gradient of caudal expression. Evidence suggests that *D. melanogaster *eIF4E-2 binds specifically to a region of bicoid resembling the eIF4E-binding region within eIF4G. This suggests that inhibition of caudal mRNA translation is due to sequestration of the caudal mRNA into a inactive 'circular' complex with which eIF4E-1 and ribosomes cannot interact. Such a mechanism of translational regulation through eIF4E-2 may not be restricted to *D. melanogaster. *The nematode Class II representative, IFE-4, is expressed in *C. elegans *in pharyngeal and tail neurons, body wall muscle, spermatheca and vulva, suggesting a special use [30]. Reduction of IFE-4 expression by RNA-interference or introduction of a null mutation produces a pleiotropic phenotype that includes an egg laying defect. Microarray analyses of mRNAs translated in the absence of IFE-4 expression suggest that IFE-4 is required for translation of a subset of mRNAs [28,30]. In mammals, expression from the *H. sapiens *eIF4E-2A gene (EIF4EL3) is upregulated following conversion of primary solid tumors to associated metastases [49] further suggesting a regulatory role for this protein.

Evidence for the expression of two distinct sub-forms of Class II eIF4E-family members can be recognized in the Actinopterygii (*D. rerio*, *O. mykiss*, and *T. rubripes*) and Amphibia (*A. mexicanum*, *X. laevis *and *X. tropicalis*) (see species containing both eIF4E-2A and eIF4E-2B in Figure 2, and data not shown). The actinopterygian and amphibian sub-forms termed eIF4E-2A represent orthologues of mammalian eIF4E-2A. The sub-forms termed eIF4E-2B are ~85–90 % identical within the core regions to eIF4E-2A sub-forms from the same species. Examination of the genomes of *H. sapiens *and *M. musculus *fails to reveal genes corresponding to a mammalian eIF4E-2B sub-form.

### Class III eIF4E-family members

**Figure 8:**
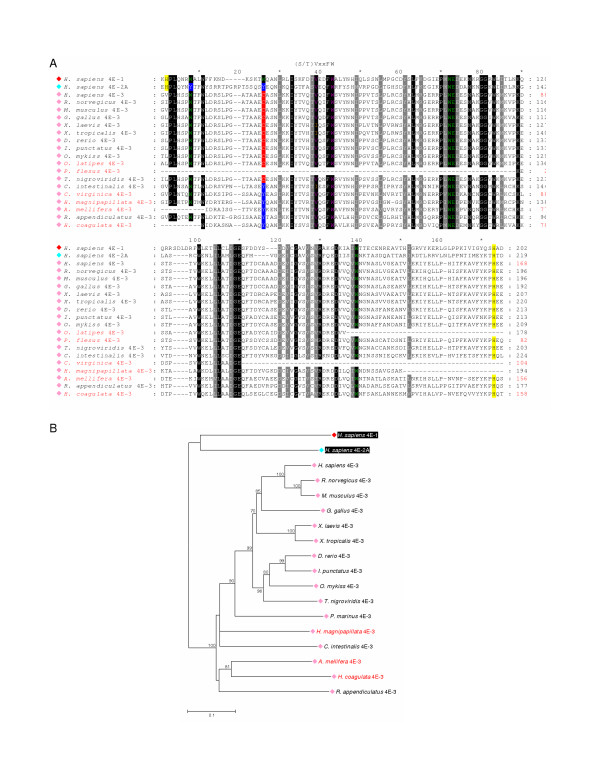
**Comparison of the conserved core regions of selected Class III eIF4E-family members**. **A. **An alignment of amino acid sequences representing the conserved core regions of the Class III eIF4E-family members from the taxonomic species indicated and of *H. sapiens *eIF4E-1. Amino acid residues within the alignment are highlighted as described in the legend to Figure 3A. Numbers to the right of the alignment represent distances of amino acids with respect to the N-terminal Met residue (black) or, for eIF4E-family members for which the N-terminal Met could not be predicted, from the first residue shown (red). **B. **A phylogram constructed by neighbor-joining derived from an alignment of nucleotide sequences representing the conserved core regions of the eIF4E-family members indicated. Bootstrap values greater than 70% derived from 50,000 tests are shown to indicate supported nodes. For **A **and **B**: names of eIF4E-family members in red indicate that only a portion of the conserved core region could be predicted.

An alignment of identified Class III proteins (sub-group 7 in Figure 2) and their relationships is presented in Figure 8A and 8B. Class III members share ~25–30% identity and ~45–55% similarity with members from Class I and Class II (Figure 3B and data not shown). Structural Class III members possess a Cys (in Vertebrata) or Tyr residue at the position equivalent to Trp-56 of *H. sapiens *eIF4E-1. Unlike Class II eIF4E-family members from Viridiplantae and Metazoa, but similar to all Class I members and Class II members from Fungi, all identified members of Class III possess a Trp residue equivalent to Trp-43 of *H. sapiens *eIF4E-1. The Class III members recognized have only been identified in Metazoa. They are well represented in chordates from tunicates, agnathans, jawed fish and higher vertebrates. However, Class III eIF4Es are sporadically represented elsewhere in Metazoa, being found in Cnidaria (*Hydra*) and in some molluscs (*Crassostrea virginica*) and some insects and arachnids. No evidence supporting the expression of Class III eIF4E-family members was found in nematodes. In Insecta, Class III eIF4E-family members can be found in Pterygota, the winged insects, with representatives from Hemiptera (sharpshooter, *Homalodisca coagulata*) and Hymenoptera (honey bee, *Apis mellifera*). However, genes encoding Class III eIF4E-family members are absent in Diptera such as *D. melanogaster *or *Anopheles gambiae*. In Arachnida, a Class III eIF4E-family member can be identified in the Parasitiforms (brown ear tick, *Rhiphicephalus appendiculatus*). The origins of Class III may result from a early duplication of a Class II gene as suggested in Figure 2A. A fuller picture of its phylogenetic distribution and evolutionary relationships will depend on the accumulation of more sequence data from more non-chordate metazoa. However, it seems possible that a progenitor Class III eIF4E-gene evolved early in metazoan evolution, but was subsequently lost in some groups.

Class III eIF4E-family members from Vertebrata possess a non-aromatic Cys residue at the position equivalent to Trp-56 of *H. sapiens *eIF4E-1. Since Trp-56 together with Trp-102 of *H. sapiens *eIF4E-1 partakes in π-bond stacking interactions with the guanine base of the cap-structure, the ability of vertebrate eIF4E-3 to interact with the cap structure would be unexpected. However, studies with *M. musculus *eIF4E-3 have shown that the protein does interact with the cap-structure *in vitro *suggesting that stacking of only one aromatic residue is sufficient for cap-interaction [26]. However, the interaction of *M. musculus *eIF4E-3 with the cap-structure is weaker than that of either mammalian eIF4E-1 or eIF4E-2. Furthermore, *M. musculus *eIF4E-3 is less able to distinguish between 7-methylated and non-methylated GTP. *M. musculus *eIF4E-3 can interact with eIF4G but not with 4E-BPs suggesting that it may participate in translation. However, *M. musculus *eIF4E-3 is unable to rescue the growth of *S. cerevisiae *lacking a functional eIF4E-gene. The weaker interaction of *M. musculus *eIF4E-3 with the cap-structure relative to eIF4E-1 suggests that this protein may be involved in sequestration of eIF4G resulting in inhibition of cap-dependent translation. The distribution of eIF4E-3 in adult mice differs from that of both eIF4E-1 and eIF4E-2, with highest levels of expression in skeletal muscle.

### eIF4E-family members from some species of Protista show extension or compaction relative to Class I, II, and III eIF4E-family members

**Figure 9:**
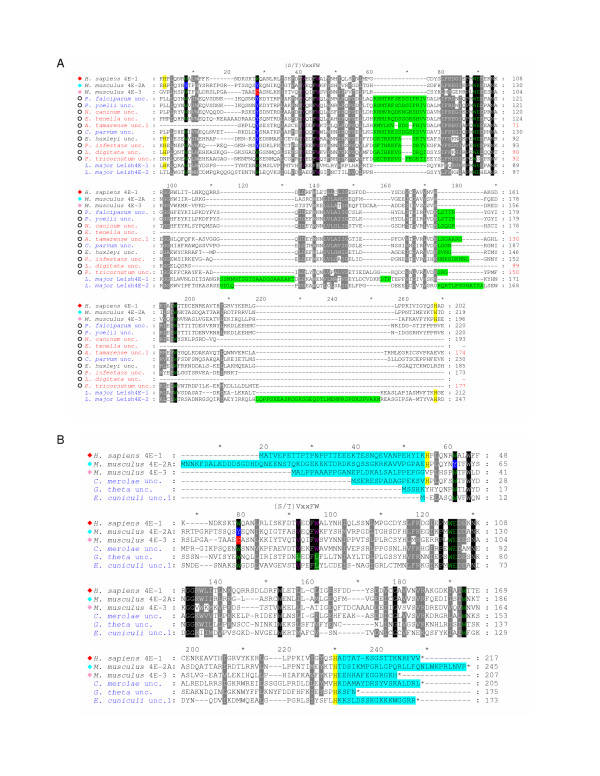
**eIF4E-family members from some species of Protista show extension or compaction**. **A. **An alignment of amino acid sequences representing the conserved core regions of eIF4E-family members from Alveolata, Stramenopiles, the Haptophyceae *E. huxleyi *and of *H. sapiens *eIF4E-1, and *M. musculus *eIF4E-2A and eIF4E-3. Green boxes indicate amino acids extensions relative to Class I, II, or III eIF4E-family members from other species. **B. **An alignment of the complete predicted amino acid sequences of predicted eIF4E-family members from *C. merolae*, *G. theta *nucleomorph, and *E. cuniculi*, and from *H. sapiens *eIF4E-1, and *M. musculus *eIF4E-2A and eIF4E-3. Residues shaded in light blue indicate regions N- and C- terminal to the conserved core of the respective eIF4E-family member. Residues shaded in greenindicate variations at positions equivalent to Val-69 and Trp-73 of *H. sapiens *eIF4E-1. For both **A **and **B**: amino acid residues within the alignment are highlighted as described in the legend to Figure 3A with the exception that residues shaded in grey indicate amino acids similar in greater than 80% (**A**) or 70% (**B**) of the sequences shown. Numbers to the right of the alignments represent distances of amino acids with respect to the N-terminal Met residue (black) or, for eIF4E-family members for which the N-terminal Met could not be predicted, from the first residue shown (red). eIF4E-family members for which names are shown in red indicate that only a portion of the core region for that member could be estimated. eIF4E-family members for which names are shown in blue indicate that sequences were predicted using genomic sequence data.

The amino acid sequences representing the core-regions or complete sequences of some of the identified eIF4E-family members from several unicellular eukaryotes are presented in Figure 9A and 9B. Examination of the sequences reveal that they differ significantly from typical Class I, Class II, or Class III eIF4E-family members.

eIF4E-family members of the Alveolata, Stramenopiles, and Haptophyta (presented as those of sub-group 8 in Figure 2) possess ~20 % identity and ~40 % similarity with respect to the core regions of eIF4E-family members from Class I, II and III (Figure 3B, and data not shown). Members of sub-group 8 possess either a Trp or Tyr residue equivalent to Trp-56 of *H. sapiens *eIF4E-1 and a Trp at the residue equivalent to *H. sapiens *Trp-43 (Figure 9A). Consequently, sub-group 8 members have Class I, Class II (fungal), or Class III-like signatures. However, members of sub-group 8 also possess extended stretches of 12–15 amino acids between residues equivalent to Trp-73 and Trp-102 of *H. sapiens *eIF4E-1, and 4–9 amino acids between residues equivalent to Trp-102 and Trp-166. Such stretches, the purpose of which are not known, are not seen in any family members of Class I, II and III and suggest that sub-group 8 members have specialized functions relative to those of Class I, II and III eIF4Es. Consequently, subgroup 8 members could be considered a fourth Class of eIF4E-family member.

A clue as to the possible role of extended sequences in the basic structure of eIF4E arises from the studies of eIF4E-family members from the trypanosome *Leishmania major. *Four intron-less eIF4E-family member genes can be identified from the known genomic sequences of *L. major *(data not shown). The sequences of two of these members (Leish4E-1 and Leish4E-2) are also presented in Figure 9A. Leish4E-1 and Leish4E-2 possess a Class I-like signature (Trp-residues at the equivalent of *H. sapiens *eIF4E-1 residues 43 and 56). Like family members of sub-group 8, both Leish4E-1 and Leish4E-2 contain extended amino acid stretches between structural units of the core, although the positions or lengths of the extensions differ from those found in sub-group 8 eIF4Es. In Trypanosomatidae, polycistronic pre-mRNA transcripts are processed to generate monocistronic RNAs which are further modified by the addition of capped spliced leader (SL) RNA. Unlike the tri-methylated SLRNAs from nematodes, the SLRNA of Trypanosomatidae possess mono-methylated cap-structures [50] that are further modified on the first four transcribed nucleotides by addition of 2'-O methyl groups resulting in all mRNAs containing so-called cap-4 structures [59]. The presence of cap-4 and/or the nucleotide sequence of the SLRNA is thought to be required for efficient recruitment of trans-spliced mRNAs into polysomes [51]. Studies *in vitro *have shown that *L. major*, LeishIF4E-1, which contains two areas of extended sequence between the structural units of the core, binds both m^7^GTP and the cap-4 structure with similar affinities [34]. This is in contrast to mammalian eIF4E-1 which possesses a 5-fold greater affinity for m^7^GTP compared to cap-4. Although trans-splicing has not been demonstrated in Alveolata, Stramenopiles, or Haptophyta, the presence of extensions within the core regions may signify that these eIF4E-family members, like Leish4E-1, recognize more complex cap-structures, specific modifications, or other sequences close to the cap-structure of a mRNA. This is of particular interest because many representatives of these sub-kingdoms are parasites or infectious agents.

Although reliant only on genomic data, specialization of a different nature can be seen in eIF4E-family members from the microsporidian, *Encephalitozoon cuniculi*, and of the algal endosymbiont of the cryptophyte *Guillardia theta *(Figure 9B). The diminutive genome of the *G. theta *algal endosymbiont (~0.55 Mb [52]) has undergone extreme compaction relative to the genomes of other Rhodophyta such as *Cyanidioschyzon merolae *which has a genome of ~16.5 Mb [53]. The *E. cuniculi *genome is also highly compacted at ~2.9 Mb [54]. Consistent with compaction, predicted Class I-like eIF4E-family members from both possess short N-and/or C-termini relative to eIF4E-family members from other species.

Trp-73 of *H. sapiens *eIF4E-1 has been shown to be involved in the interaction of eIF4E-1 with eIF4G and 4E-BPs. With the exception of *D. melanogaster *eIF4E-1d, all members of the three defined non-protist structural classes of eIF4E-family members possess a Trp-residue equivalent to Trp-73 of *H. sapiens *eIF4E-1. Examination of the eIF4G-binding regions of eIF4E-family members from both *E. cuniculi *and the *G. theta *nucleomorph reveals differences relative to *H. sapiens *eIF4E-1. In both cases, residues equivalent to Trp-73 of *H. sapiens *eIF4E-1 are substituted by a non-aromatic Leu residue. As discussed earlier, such a substitution in *H. sapiens *eIF4E-1 has been shown to impair the ability of eIF4E-1 to interact with either eIF4G or with 4E-BPs [20,21]. In the case of the *G. theta *algal endosymbiont this variation may not be remarkable since the genome of the nucleomorph appears to lack any identifiable sequence encoding eIF4G-like or 4E-BP-like proteins. Since the genome of the *G. theta *endosymbiont has been so severely compacted, it is hypothesized that the genes encoding complex cellular functions in the endosymbiont, such as protein synthesis, are limited to the minimal set needed to accomplish the function. Although the endosymbiont has its own mRNAs with 5'-caps and poly(A) tails, elongation and release factors, its genome only encodes a subset of translational initiation factors: eIF1, eIF1A, eIF4A, eIF2 (all subunits, although the alpha subunit is truncated), eIF4E, eIF6 and poly(A) binding protein. The genes for many initiation factors thought to be essential for cannot be identified including eIF4B, eIF5, or the scaffold protein eIF3 (any subunit).

## Conclusion

eIF4E, a translational initiation factor found only in eukaryotes has a unique alpha/beta fold that is considered to have no homologues outside the eukaryotes, as determined by sequence comparison or structural analyses [55]. The expansion of sequenced cDNAs and genomic DNAs from organisms of all taxonomic kingdoms has significantly altered our picture of eIF4E which must now be considered to be one member of a structurally related family of eIF4E-like proteins.

Evolutionarily it seems that a single early eIF4E gene has undergone multiple gene duplications generating multiple structural classes. The functions of each member of each structural class remain to be completely understood. However, it is no longer possible to predict eIF4E-function from the primary amino acid sequence of an eIF4E-family member, as exemplified by the functional diversity of examples mentioned here and recently reviewed elsewhere [27]. The ancestral gene of eIF4E has also provided a blueprint for the generation of related proteins with specialized functions found only in certain taxonomic groups. The *C. elegans *IFE-1, -2 and -5 appear to result from gene duplications that occurred within early nematodes to give rise to a specialized sub-class that recognizes alternate cap structures. No direct relatives of these tri-methyl-cap binding proteins can be found in any other phyla. The extended eIF4Es of certain protists, like *L. major *have evolved independently to fulfill a similar function. These eIF4E variants seem likely to provide a rich source of variations on the eIF4E structural theme that will provide unique opportunities for structure/function studies and therapeutic drug design. As more sequence data becomes available and more eIF4E-family members are tested for their activities both *in vitro *and *in vivo *our understanding of the origins and functions of individual members will advance. The data provided here have been deposited in an internet accessible database for online access to assembled sequences encoding eIF4E-family members [35]. The site has been developed to allow easy searches, as well as sequence comparisons and other analyses.

## Methods

### Acquisition of cDNA sequences encoding eIF4E-family members

The nucleotide sequences encoding *M. musculus *eIF4E-1, eIF4E-2, and eIF4E-3, *T. aestivum *eIF4E and eIF(iso)4E, *A. thaliana *nCBP, *C. elegans *IFE-1, 2, 3, 4, and 5, and *S. cerevisiae *eIF4E were used to probe GenBank (NR), and dbEST databases for homologous cDNA sequences from other species through use of the BLAST 2.0 software package. In an iterative process, the retrieved sequences were used to re-probe the databanks to obtain further sequences of overlapping cDNA fragments from the same organism or to obtain related sequences from additional species. For budding yeasts and some protists, which lack, or possess few numbers of, introns in genes transcribed from RNA polymerase II promoters, genomic sequences encoding eIF4Es were also acquired from the GenBank database. The expression of many of these genomic sequences have been verified by the presence of at least one EST sequence for each.

### Derivation of consensus cDNA sequences encoding eIF4E-family members

Overlapping nucleotide sequences encoding an eIF4E-family member from a particular species were aligned to produce complete or partial consensus cDNA sequences. In most cases, sequences within 3'-UTRs were used to verify that EST sequences described the same eIF4E-family member. However, due to usage of alternative splicing and/or of alternative polyadenylation sites, 3'-UTRs sometimes differed in sequence. Sequences were not considered verified unless a minimum of two sequences representing overlapping cDNA fragments confirmed the assignment of a single nucleotide. Where multiple nucleotides assignments for a particular base in a consensus sequence were supported by multiple sequences these variations were considered to represent polymorphisms or variations in strains. To allow for such variations, information about the strain used for development of the cDNA library from which an EST was recovered (provided by some submitters to GenBank databanks) was utilized to select a subset of sequences. Where strain information was not available, or where strain information still suggested multiple possibilities for an assignment, the assignment of a base supported by the majority EST sequences was chosen. In almost all cases these variations led to either no change at the amino acid level due to codon-degeneracy, or a single amino acid variation. Furthermore, such variations were either confined to regions encoding segments N-terminal of the core of the eIF4E-family member or had no affect on the prediction of amino acids in positions related to those known to be involved in the interaction of mammalian eIF4E-1 with the cap-structure, eIF4G, and 4E-BPs.

### Alignments and analyses of sequences representing eIF4E-family members

Since alignments of mammalian eIF4E-1s, plant eIF4E and eIF(iso)4E and *S. cerevisiae *eIF4E suggest the presence of an evolutionarily conserved core region and because deletion analyses of *S. cerevisiae *eIF4E and *D. rerio *eIF4E-1A suggest that the N-termini and C-termini are dispensable with respect to cap-binding, eIF4G and 4E-BP interaction, sequences representing the core region of an eIF4E-family member were used for analyses unless otherwise stated. Amino acid alignments were performed using ClustalW (version 1.8) software and adjusted as necessary [56]. Alignments of the nucleotide sequences representing the core regions of eIF4E-family members (~480–510 nucleotides per member) were generated by reverse translation of amino acid alignments, and substitution of the factual nucleotide sequences. Concensus phylogenetic trees were generated from nucleotide alignments using the "neighbor-joining" and "boot-strapping" algorithms within Mega 3.0 [57]. In some cases, only a part of the nucleotide sequence representing the core region of an eIF4E-family member could be identified. Where indicated, partial sequences were included in the analyses. Unless stated, names assigned to eIF4E-family members were based on designations previously applied by investigators in the field of translational control.

Analyses of the consensus cDNA sequences of 220 representative eIF4E-family members from 118 species are presented. The predicted amino acid sequences, accession numbers of all sequences used to derive consensus cDNA sequences, and full names of all the species from which they were derived, are supplied in the form of Additional File 1. Sequence alignments and derived concensus cDNA sequences corresponding to all eIF4E-family members identified are available through the 'eIF4E-family member database [35].

## Authors' contributions

BJ conceived of the study, designed and constructed the eIF4E-family member database [35], acquired and aligned sequences, analyzed the sequences for their phylogenetic relationships, and drafted the manuscript. KL participated in database management and sequence alignment. DM participated in the analyses of sequences for their phylogenetic relationships. RJ conceived of the study, participated in its design and coordination, and helped draft the manuscript.

## Supplementary Material

Additional File 1 List of species names, predicted amino acid sequences, and accession numbers. Click here for file
